# Supplementary figures and images for: Characterization of C9orf72 haplotypes to evaluate the effects of normal and pathological variations on its expression and splicing
Source: PLoS Genet. 2021 Mar 29;17(3):e1009445. doi: 10.1371/journal.pgen.1009445 (PMC8031855; doi:10.1371/journal.pgen.1009445)

## *C9orf72* variants

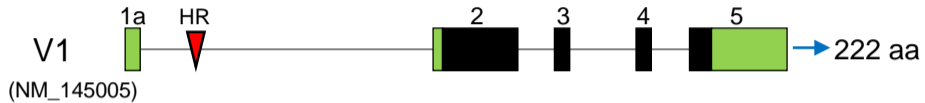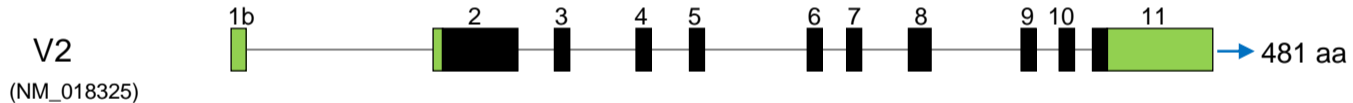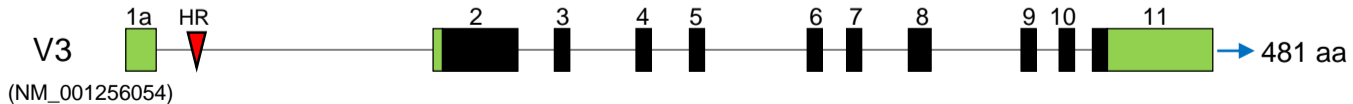

Supplement: S1 Fig — C9orf72 variants named as in PubMed and the UCSC Genome. Black and green blocks represent translated and untranslated exonic regions, respectively, and red triangles represent the HR site. (PDF) [file pgen.1009445.s001.pdf]

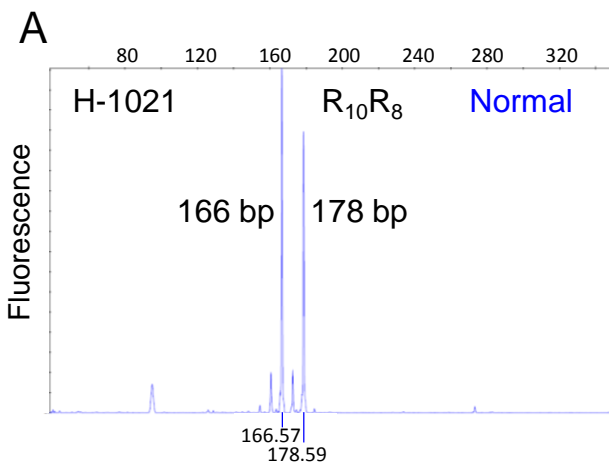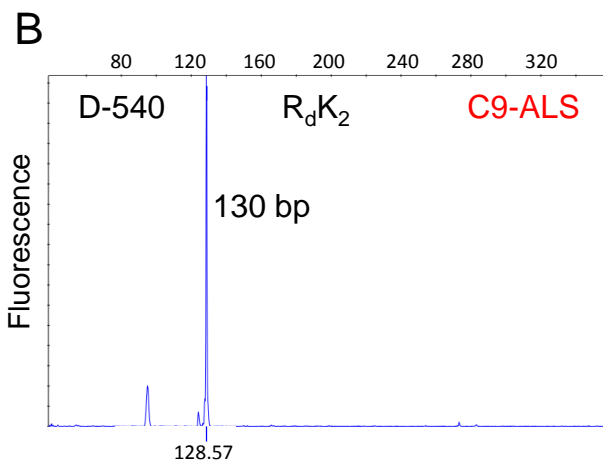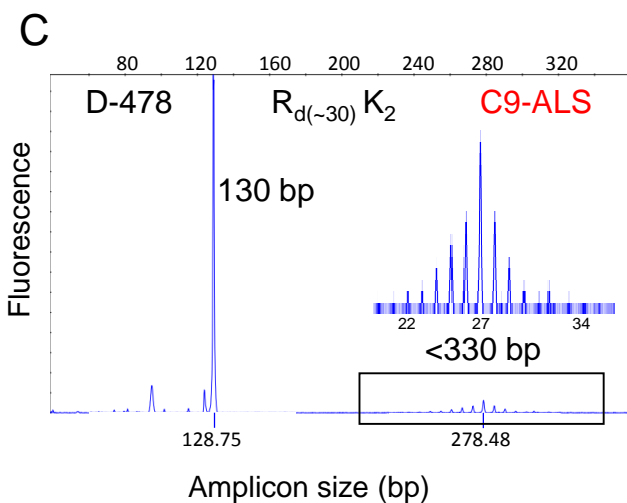

Supplement: S2 Fig — The length of each amplified product is indicated. The number of HR units was calculated by subtracting the length of the 5’ and 3’ flanking regions (total 118 bp) from the PCR product length and dividing by six. (A) PCR amplified products of control H-1021 (homozygous for Rh alleles) are 178 and 166 bp long, indicating HR repeats of 10 and 8 units (R10R8). (B) PCR amplified product of the RdK-ALS patient D-540. The 130 bp fragment reveals HR length of 2 units at the K allele. The length of the Rd allele in D-540 was previously determined by Southern blot analysis as 350–390 repeats and is not PCR amplified. (C) PCR amplified product from RdK ALS patient D-478. The 130 bp fragment reveals HR length of 2 units in the K allele. The R allele HR length appears as sawtooth peaks of 20–35 repeats and a maximal peak of 27 repeats. (PDF) [file pgen.1009445.s002.pdf]

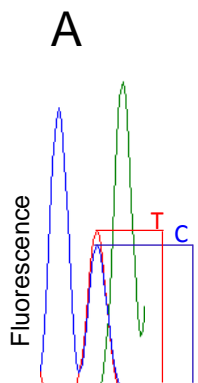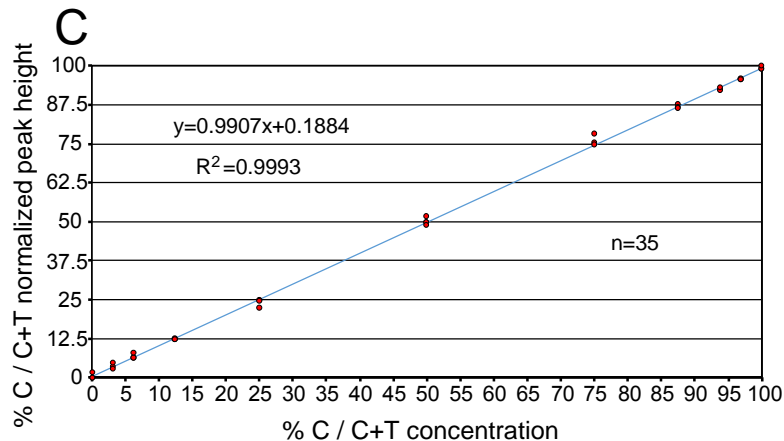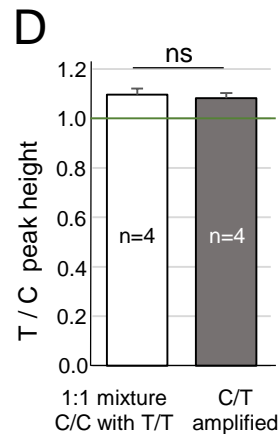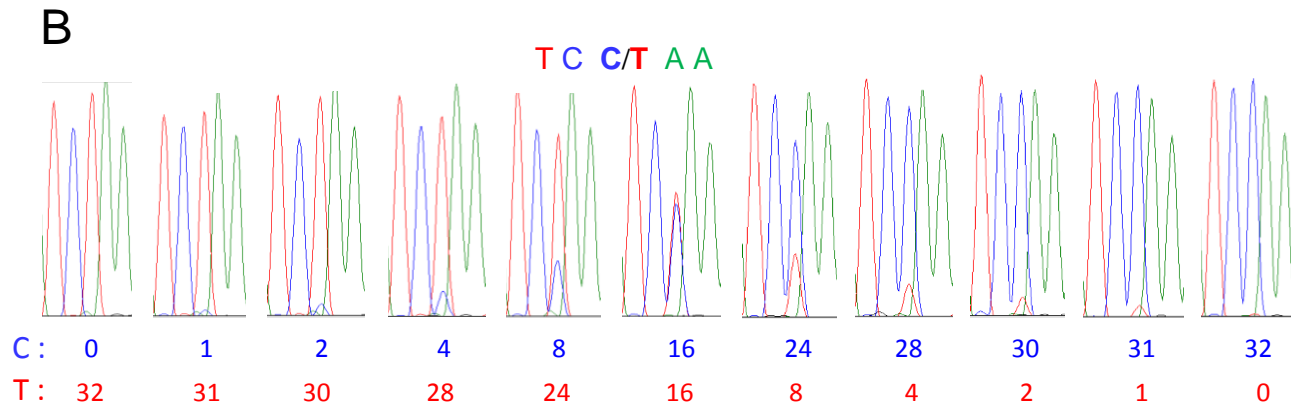

Supplement: S3 Fig — (A) Representative Sanger sequencing chromatogram of equimolar amounts of two 659 bp DNA fragments that differ in a single nucleotide (C versus T in rs10757668; S1 Table, #11). The peak height of T is higher than C. The PCR fragments originated from H-1021 and H-1116 fibroblasts that carry RR and KK haplotypes, respectively (S3 Table). (B) Representative Sanger sequencing chromatograms of different mixtures of two 659 bp long DNA fragments (S1 Table, #11) that differ in a single nucleotide (C versus T). (C) Mixture series of opposite homozygous DNA fragments as in (B) demonstrate a linear relationship between the relative allelic contribution and the relative peak height. Peak-height ratios (C/C+T) were normalized according to the 1:1 allelic ratio shown in (A) and plotted against the actual concentration ratios. The plot shows 35 data points obtained from three or four independent sequencing runs for each mixture. Some points are co-localized and appear as a single point. The points are fitted with best-fit linear regression line and shown together with the R2 value and the calculated equation for the slope. (D) T/C peaks height ratio in equimolar mixture of opposite homozygous DNA fragments as in (A) compared to PCR amplification of heterozygous genomic DNA. The peak height ratio in the two groups is similar. ns: non-significant. (PDF) [file pgen.1009445.s003.pdf]

# C9-ALS iPSCs - Intron 1

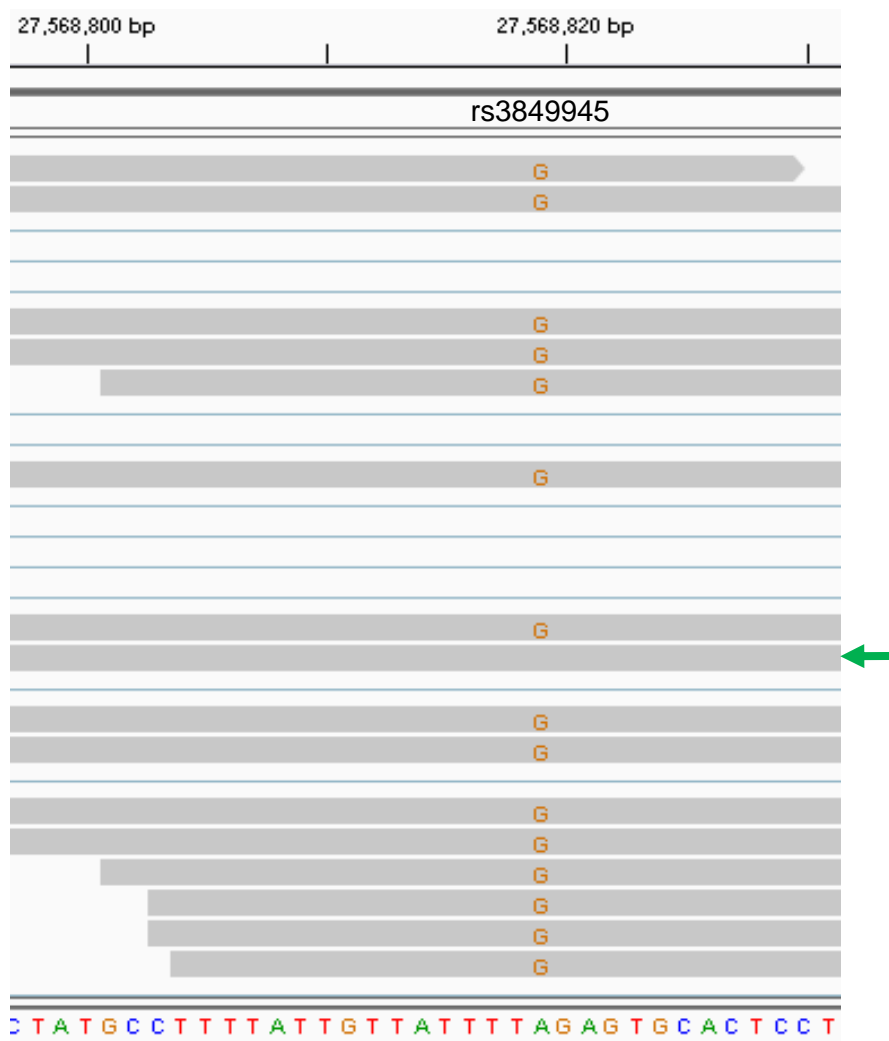

Supplement: S4 Fig — Representative RNA-seq reads from C9-ALS iPSCs (D-312). The reads spanning the heterozygous position of SNP rs3849945 in the first intron of C9orf72 gene. Thirteen of the identifiable reads originated from the Rd allele and carry G nucleotide (orange), while only one carry the reference A nucleotide (indicated by green arrow), which originated from the complementary F allele. (PDF) [file pgen.1009445.s004.pdf]

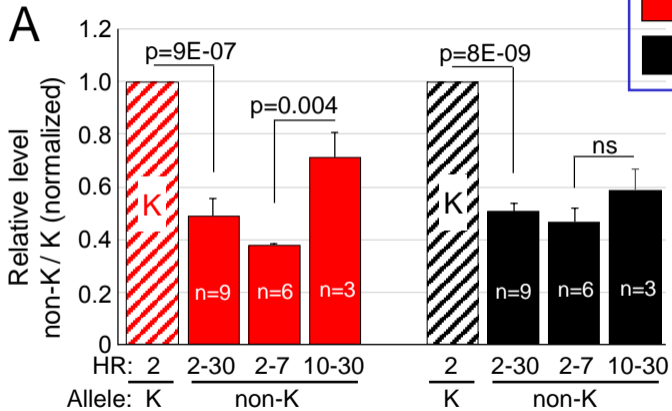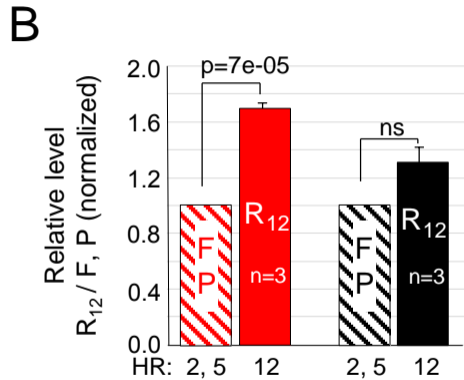

Supplement: S5 Fig — (A) Allelic analysis of introns 1 and 2 in normal and intermediate fibroblasts that carry K allele and a complementary allele with 2-~30 repeats (n = 9). The ratio in the intronic SNPs was normalized to the ratios obtained in exons 2, 5, and 11 (S1 Table, #12, 24 and 38). The dashed columns represent the expression levels of the K alleles and the left solid columns represent the relative expression of the complementary alleles. Analysis according to the length of the non-K allele reveals higher accumulation of intron 1 in lengths of 10-~30 versus 2–7 repeats. (B) Allelic analysis of introns 1 and 2 in normal R12F and R12P fibroblasts. The ratio in intronic SNPs was normalized to the ratios obtained in exons 2, 5, and 11 (S1 Table, #12, 24 and 38). The contribution of R12 allele to introns 1 is higher than the complementary F and P alleles that carry 2 and 5 repeats, respectively. Data presented as mean ± SEM. ns: non-significant. (PDF) [file pgen.1009445.s005.pdf]

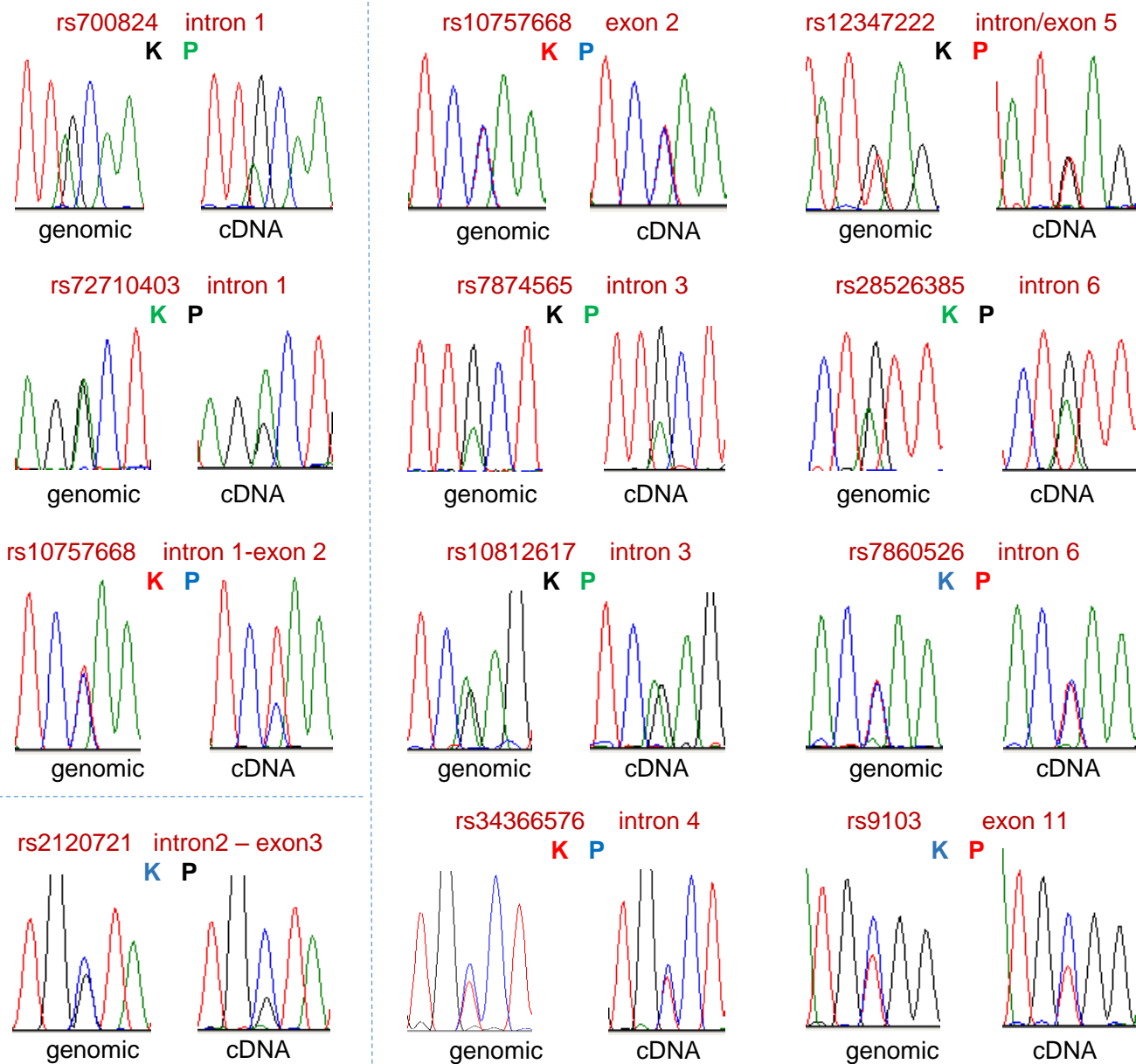

Supplement: S6 Fig — A representative set of Sanger sequencing chromatograms for different regions of the C9orf72 gene. All chromatograms represent amplification products from control H-966 fibroblasts that carry the K and P alleles (S3 Table). Each chromatogram in the figure includes five sequential nucleotides with a central polymorphic nucleotide. The alternative nucleotide color of K and P alleles is indicated by a colored letter above the chromatograms. For each chromatogram pair, the left and the right panels represent amplification products of genomic DNA and cDNA, respectively. All SNPs shown in the figure are described in S2 Table. The PCR primers used, and amplicon lengths are described in S1 Table. The relative level of the K allele is markedly higher in introns 1 and 2 but not in other introns, nor in exons. The K allele also contributed more to the amplified products that spanned intron 1-exon 2 and exon 2-intron 3 boundaries. Therefore, the relative increase in the K allele probably attributed to a reduction in its splicing efficiency. (PDF) [file pgen.1009445.s006.pdf]

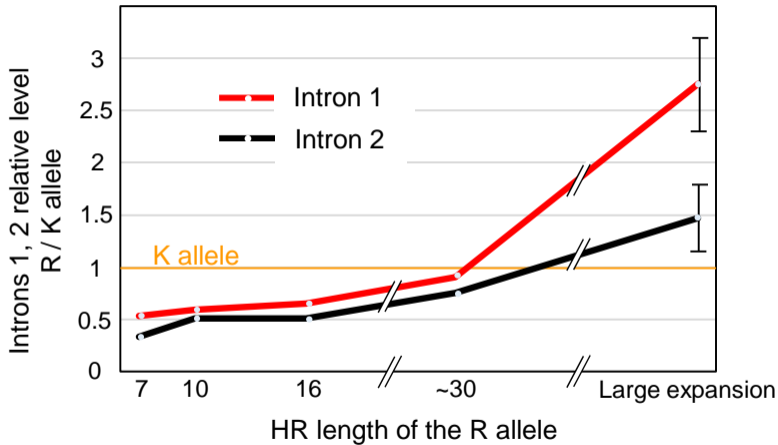

Supplement: S7 Fig — Fibroblasts that carry both R and K alleles but differ in the HR length of the R allele were analyzed for R/K allelic ratio in introns 1 (red) and intron 2 (black). The orange horizontal line delineates a ratio of 1 and represents the K allele intronic levels. (PDF) [file pgen.1009445.s007.pdf]

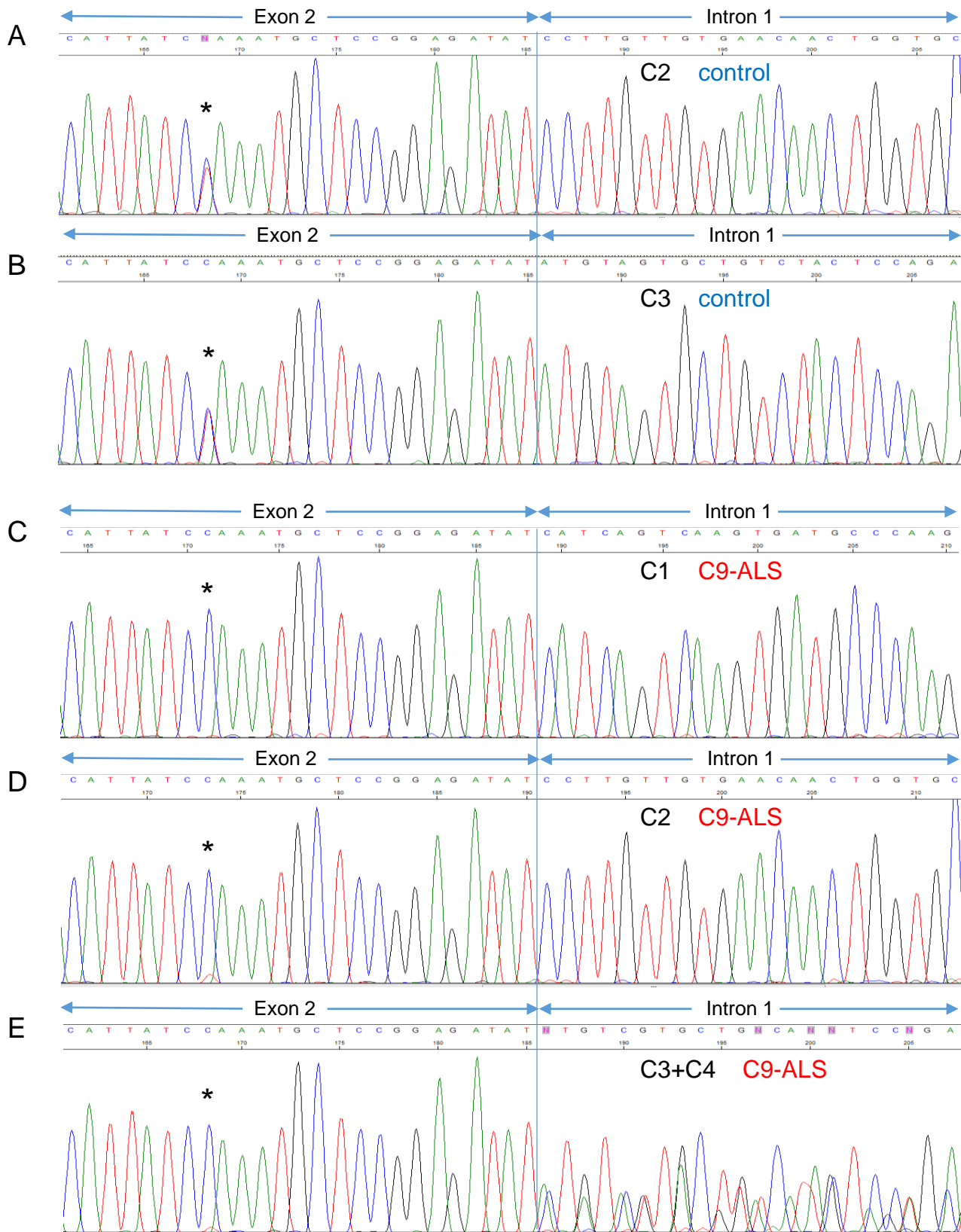

Supplement: S8 Fig — Fibroblast of control donors (A, B) and C9-ALS patients (C-E) were RT-PCR amplified for cryptic splice site products (C1-C4) and Sanger sequenced with a reverse primer in exon 2. The representative chromatograms in the figure show the complementary strand of the products described in Fig 4A. All cells carry a single K haplotype and therefore are heterozygous for rs10757668 (C/T), marked by an asterisk. In normal cells, the relative contribution of two normal alleles is frequently similar (A, B); while in C9-ALS cells, the vast majority of C1-C4 transcripts are the Rd allele products. C3 and C4 were co-amplified in (E). (PDF) [file pgen.1009445.s008.pdf]

Control iPSCs

A

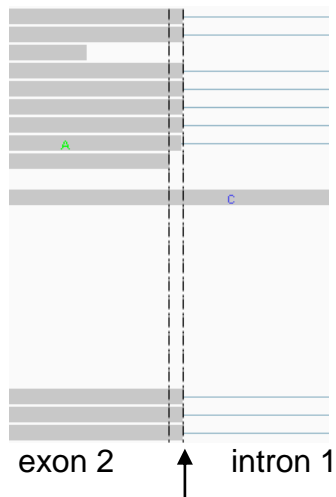

C9-ALS iPSCs

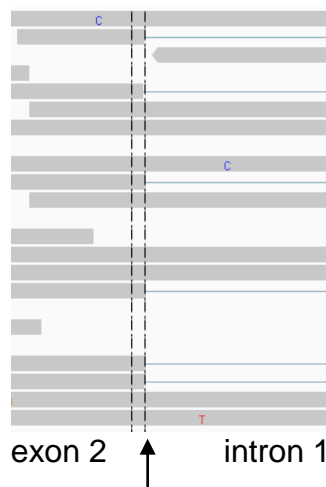

C9-ALS iPSCs

B

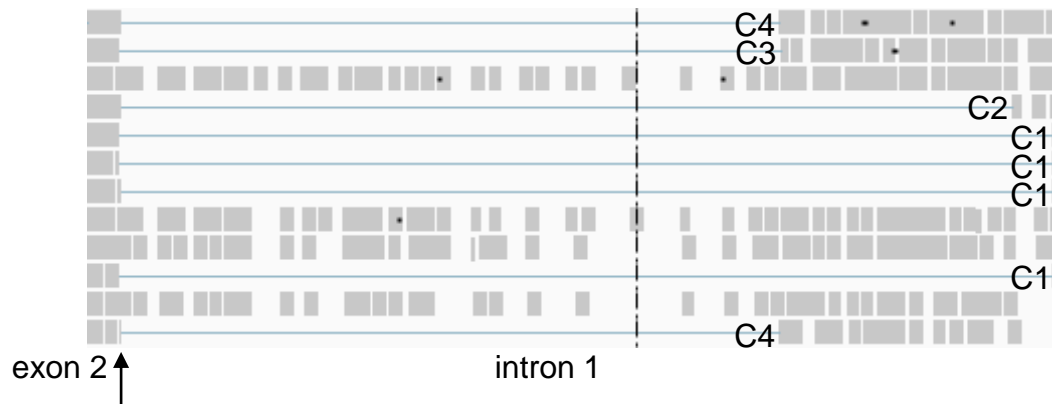

Supplement: S9 Fig — (A) RNA-seq mapping results for intron 1-exon 2 boundary region. The frequency of reads spanning intron 1-exon 2 junction (unspliced) is higher in C9-ALS than in control iPSCs. (B) RNA-seq mapping results in a representative C9-ALS iPSC line. Reads overlapping cryptic splice junction are indicated (C1-C4). Black arrows indicate the boundary between intron 1 and exon 2. (PDF) [file pgen.1009445.s009.pdf]

# A

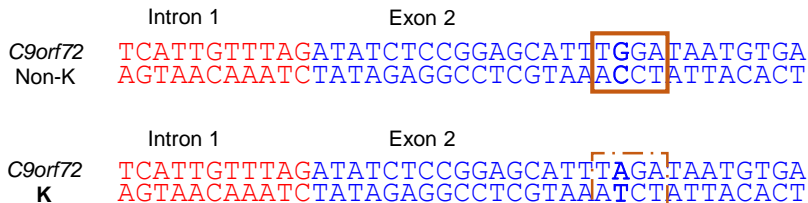

# B

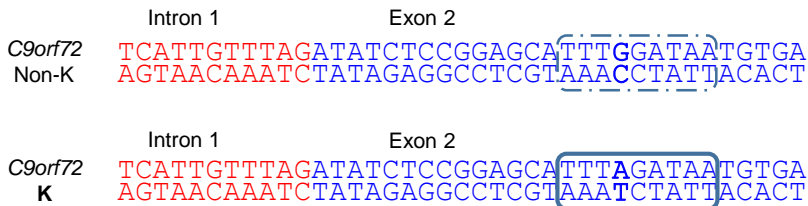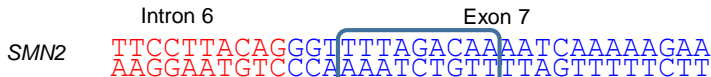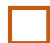

Exonic splicing enhancer

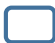

Exonic splicing silencer

Supplement: S10 Fig — Substitution of G to A in SNP rs10757668 (TTTGGATAA to TTTAGATAA) is specific to K haplotype. This SNP is in exon 2, just 18 bp downstream of intron 1. (A) It may account for destroying a putative TGGA exonic splicing enhancer site [47]. (B) Alternatively, it may account for the formation of exonic splicing silencer site resembling the hnRNP A1 binding site (TTTAGACAA) in the SMN2 gene [46]. (PDF) [file pgen.1009445.s010.pdf]

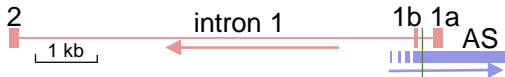

Control

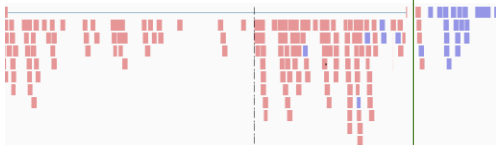

C9-ALS

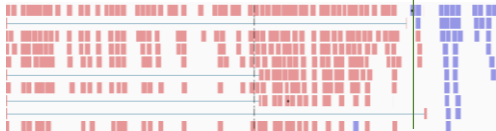

Supplement: S11 Fig — Examples for strand-specific RNA-seq analysis of control and C9-ALS cells. Reads of sense and antisense (AS) transcripts in the 5’ region of the gene C9orf72 gene (~8 kb) are illustrated in pink and blue, respectively. The green line indicates the HR site. Most transcripts that are located between exon 1b and exon 2 are sense transcripts. (PDF) [file pgen.1009445.s011.pdf]
