# Supplementary material for: Characterization of C9orf72 haplotypes to evaluate the effects of normal and pathological variations on its expression and splicing
Source: PLoS Genet. 2021 Mar 29;17(3):e1009445. doi: 10.1371/journal.pgen.1009445 (PMC8031855; doi:10.1371/journal.pgen.1009445)
Supplement: S1 Table — (DOCX) [file pgen.1009445.s012.docx]

**S1 Table. PCR primer pairs used for SNP analysis in this study**

| **Product**  **Size (bp)** | **SNPs (rs#)**  **in the PCR product** | **Primers sequences** | **Target site** | **PCR**  **#** |
| --- | --- | --- | --- | --- |
|  |  | PCR for genomic DNA and unspliced mRNA |  |  |
| 651 | 2244606, 10967991, 11789520 | 5-TGTAGATCAGATGGTCTTCGGT  5-GTCTTTTGGTCTGCAGGTGT | 5' flanking | 1 |
| 298 | 41272893, 78074330 | 5-GTCCCGCTAGGAAAGAGAGGT  5-GACTCCTGAGTTCCAGAGCTTG | Exon 1a  Intron 1 | 2 |
| >130 | HR | FAM-CAAGGAGGGAAACAACCGCAGCC  5-GCAGGCACCGCAACCGCAG | Intron 1 (HR)  Intron 1 | 3 |
| 413 | 117462033, 112048460 | 5-CTCATTTCTCTGACCGAAGCTG  5-CATCACCACTCTCTAGAAGCTTGG | Intron 1 | 4 |
| 550 | 2282240, 2282241 | 5-GTGCTCATTGGGTCTATCTGG  5-GCAGGTAAATGCTGGCTTAGTG | Intron 1 | 5 |
| 792 | 4520261, 17696653, 3849946 | 5-CAGGAGGACTAGGTTAGCCTACCT  5-CTTGCTCACAGGGTTCATGAG | Intron 1 | 6 |
| 569 | 700824, 700825, 4879572 | 5-TTGCTCCAGGGTTCAGTTCTG 5-TGTCAAGAGAGTTCAGTCTGATGG | Intron 1 | 7 |
| 1023 | 76602706, 13284967,111630075  10812619, 72710405 | 5-CATAGGTGACAGCTACATGTGTG  5-CCATCAGACTGAACTCTCTTGAC | Intron 1 | 8 |
| 503 | 3849945, 72710403, 77534147 | 5-CACACATGTAGCTGTCACCTATG  5-ACGCTCACATCCAAGACAGAAC | Intron 1 | 9 |
| 809 | 7875392, 17769300, 7872223 10967988, 10757669 | 5-GTGTAGAACTCTCTTTGCAGTGATG  5-CCATGTGCAATTCTAGTATGACTGG | Intron 1 | 10 |
| 659 | 41272891, 10757668 | 5-CAAGTGTCAGTCTCTAGCTGAGC  5-TCTCCATCACTGAGAAGTACCTG | Intron 1  Exon 2 | 11 |
| 273 | 10757668 | 5-GATATCTCCGGAGCATTT  5-CTCTGCATTTCGAAGGAT | Exon 2 | 12 |
| 698 | 10757668 | 5- GATATCTCCGGAGCATTT  5-GAGCTACCATTTCGTACCT | Exon 2  Intron 2 | 13 |
| 762 | 2120721, 1031153, 10757667 | 5-TGGTCATGTAGCCTTTGGTCTC  5-CTGATCTTCCATTCTCTCTGTGC | Intron 2  Exon 3 | 14 |
| 640 | 10967986, 10757666, 10812618  142843265, 2492816 | 5-CTTAGAAGGCACAGAGAGAATGG  5-CACCCTTGTAACCATGCTTCTC | Exon 3  Intron 4 | 15 |
| 620 | 7859060 | 5-CTTTACCCAAAGAATCCCAGTC  5-TCAGATGACAGCTACAACTGAAG | Intron 3 | 16 |
| 560 | 10441712 | 5-GAGGCATGATGACTTAATCATTGC  5-ATCACTTCTCCAGTAAGCATTGGA | Intron 3  Exon 4 | 17 |
| 702 | 10812616, 10812615,  34366576, 2453554 | 5-CTGGAGAAGTGATTCCTGTAATGG  5-CACACTGCCTTCATTTCCGAG | Exon 4  Intron 4 | 18 |
| 319 | 17769294 | 5-AGTCTCCTGATTCTGACAGCTCA  5-GAGAAGAAAGCCTTCATGACAGC | Intron 4  Exon 5 | 19 |
| 436 | 774359, 12347222 | 5-GACTCCCTGGATTAATGCTGTG  5-CTCATGCCTCTGATACTCCATC | Int/Exon 5 | 20 |
| 666 | 774358, 28526385, 774357, 1565948  774356, 7860526, 2297694 | 5-AGCTCGGAAATACCAGTGTCAG  5-GAGATCACAGATGGAAAGCCAC | Intron 6 | 21 |
| 477 | 67245195, 10757665, 4879564  112616482 | 5-TTTGAGTCTACTGTCAAGGAGC  5-TCTAGGCATCTGGTATAGGTCC | Intron 7 | 22 |
| 351 | 1022902 | 5-GCACACACTCATGAATAGCTTC  5-GTGTAGATGATCGTATCCTGAGC | Intron 7  Exon 8 | 23 |
| 613 | 3739526, 13691, 9103 | 5-AGTTCAGATTTCACTGGTCAGTC  5-CAGTCATCTAGTTCAGTGGTTGTC | Exon 11 | 24 |
| 781 | 700791, 10812612, 812858 | 5-CATCTGCAAAGCTAGGAATAGG  5-ACAAGCTGGAGATTGTCAGA | 3' flanking | 25 |
| 315 | 3849943, 3849942 | 5-GAAGAGTGGAACAGAATCTGC 5-ACCATGCTAGGCACTGAGA | 3' flanking | 26 |
|  |  | RT-PCR for mRNA splicing products |  |  |
| 402/480 | 10757668 | 5-CCCACGTAAAAGATGACGCTTG  5-TAGTCCATATGTGCTGCGATCC | Exon 1a (V1+ V3) Exon 2 | 27 |
| 728/806 | 10757668, 17769294 | 5-CCCACGTAAAAGATGACGCTTG  5-GAGAAGAAAGCCTTCATGACAGC | Exon 1a (V1+ V3) Exon 5 | 28 |
| 1421/1499 | 774359, 12347222 | 5-CCCACGTAAAAGATGACGCTTG  5-GGAACTAACATGTAGGCACTCAAC | Exon 1a (V1+ V3) Int/Exon5 | 29 |
| 393 | 10757668 | 5-CGGTGGCGAGTGGATATC  5-TAGTCCATATGTGCTGCGATCC | Exon 1b (V2)  Exon 2 | 30 |
| 720 | 10757668, 17769294 | 5-CGGTGGCGAGTGGATATC  5-GAGAAGAAAGCCTTCATGACAGC | Exon 1b (V2)  Exon 5 | 31 |
| 1411 | 774359, 12347222 | 5-CGGTGGCGAGTGGATATC  5-GGAACTAACATGTAGGCACTCAAC | Exon 1b (V2)  Int/Exon 5 | 32 |
| 932 | 117462033, 112048460 10757668 | 5- CTCATTTCTCTGACCGAAGCTG  5-TAGTCCATATGTGCTGCGATCC | Intron 1 (C1)  Exon 2 | 33 |
| 448 | 10757668 | 5-AGCTCCGAGATGACACAGACTTG  5-TAGTCCATATGTGCTGCGATCC | Intron 1 (C1)  Exon 2 | 34 |
| 585 | 2282240, 10757668 | 5-GTGCTCATTGGGTCTATCTGG  5-TAGTCCATATGTGCTGCGATCC | Intron 1 (C2)  Exon 2 | 35 |
| 403 | 10757668 | 5-GCACCAGTTGTTCACAACAAGG  5-TAGTCCATATGTGCTGCGATCC | Intron 1 (C2)  Exon 2 | 36 |
| 1258/1271 | 4520261, 17696653, 3849946, 10757668 | 5-CAGGAGGACTAGGTTAGCCTACCT  5-TAGTCCATATGTGCTGCGATCC | Intron1 (C3/4)  Exon 2 | 37 |
| 289 | 17769294 | 5-CCCACTTCATAGAGTGTGTGTTGA  5-GAGAAGAAAGCCTTCATGACAGC | Exon 2  Exon 5 | 38 |
| 786 | 774359, 12347222 | 5-GAAATCACACAGTGTTCCTGAAG  5-GGAACTAACATGTAGGCACTCAAC | Exon 4  Int/Exon 5 | 39 |
