# Supplementary material for: Characterization of C9orf72 haplotypes to evaluate the effects of normal and pathological variations on its expression and splicing
Source: PLoS Genet. 2021 Mar 29;17(3):e1009445. doi: 10.1371/journal.pgen.1009445 (PMC8031855; doi:10.1371/journal.pgen.1009445)
Supplement: S2 Table — (DOCX) [file pgen.1009445.s013.docx]

**S2 Table. Detailed genetic map of the *C9orf72* locus**

|  | **Haplotype**  **R F K P N J Q Z** | | | | | | | | **MAF**  **World EUR** | **Sequence of**  **(+) strand** | **Chrom. 9**  **Position** | ***C9orf72***  **location** | **SNP ID rs#** |
| --- | --- | --- | --- | --- | --- | --- | --- | --- | --- | --- | --- | --- | --- |
| C | | C | C | C | C | ***T*** | C | C | T=14.9% 25.0% | TGCTG[C/T]AACTG | 27574805 | 5' flanking | 10967991 |
| C | | C | C | C | C | C | C | ***T*** | T=16.2% 24.3% | ACTTA[C/T]TACAG | 27574517 | 5' flanking | 11789520 |
| G | | G | G | G | G | G | ***-*** | G | - =25.3% 25.7% | GAGTT[G/-]TTTTT | 27574312 | 5' flanking | 5897244 |
| A | | A | A | ***G*** | A | A | A | A | G=2.8% 1.8% | GTTTG[A/G]CGCAC | 27573828 | Exon 1a | 41272893 |
| A | | A | A | ***G*** | A | A | A | A | G=6.4% 1.9% | TTGCT[A/G]CAGGC | 27573579 | Intron 1 | 78074330 |
|  | | 2 | ~6 | 4 | 5 | 2 | 2 |  |  | (GGCCCC)n | 27573534 | Intron 1 | HR |
| C | | T | C | C | T | C | C | C | T=6.7% 15.8% | GCCGC[C/T]GCCGC | 27573215 | Intron 1 | 117462033 |
| C | | C | C | ***T*** | C | C | C | C | T=2.7% 1.8% | CAGTA[C/T]CCGAG | 27573085 | Intron 1 | 112048460 |
| C | | C | C | C | C | C | ***T*** | C | T=27.9% 27.7% | GGCCA[C/T]CCCTC | 27572636 | Intron 1 | 2282240 |
| A | | A | C | C | A | C | A | C | A=43.2% 44.0% | TGTAG[C/A]AGTTT | 27572257 | Intron 1 | 2282241 |
| C | | C | C | ***T*** | C | C | C | C | T=2.8% 1.8% | TCTGG[C/T]CATTT | 27571957 | Intron 1 | 4520261 |
| T | | T | T | ***C*** | T | T | T | T | C=2.7% 1.8% | AACCG[T/C]CCACT | 27571821 | Intron 1 | 17696653 |
| A | | A | C | C | A | C | A | C | A=43.0% 43.8% | CCTCA[C/A]TTCTT | 27571460 | Intron 1 | 3849946 |
| 5 | | 5 | 5 | 5 | 5 | 5 | 5 | **6** | 6 =19.4% 23.8% | (AAAC)n | 27570875 | Intron 1 | 145319692 |
| C | | C | C | ***T*** | C | C | C | C | T=2.8% 1.8% | CCGGG[C/T]ATGGT | 27570591 | Intron 1 | 78900326 |
| T | | T | C | T | T | C | T | T | C=34.6% 30.5% | TATTG[T/C]AAACA | 27570350 | Intron 1 | 700824 |
| G | | G | ***A*** | G | G | G | G | G | A=8.2% 5.2% | GCACT[G/A]GATAT | 27570156 | Intron 1 | 700825 |
| A | | A | A | A | A | A | A/G | A | G=9.0% 6.5% | AACAC[A/G]ACGAC | 27570053 | Intron 1 | 4879572 |
| T | | T | T | ***C*** | T | T | T | T | C=2.7% 1.8% | AATGA[T/C]GCATT | 27569659 | Intron 1 | 76602706 |
| T | | T | C | C | T | C | T | C | T=43.0% 43.8% | CCATA[C/T]GGCCT | 27569574 | Intron 1 | 13284967 |
| G | | G | G | ***A*** | G | G | G | G | A=2.8% 1.8% | CATTC[G/A]TGGTA | 27569398 | Intron 1 | 111630075 |
| C | | C | T | T | C | T | C | T | C=43.1% 43.8% | CTAAG[T/C]ATTTT | 27569190 | Intron 1 | 10812619 |
| G | | G | G | G | ***A*** | G | G | G | A=4.7% 10.7% | GACAA[G/A]ATGTG | 27568969 | Intron 1 | 72710405 |
| A | | A | A | A | A | A | A/G | A | G=1.2% 1.7% | TAAAA[A/G]CCTTC | 27568952 | Intron 1 | 111653040 |
| A | | A | A | A | A | A | A | ***G*** | G=13.7% 23.9% | ATTTT[A/G]GAGTG | 27568819 | Intron 1 | 3849945 |
| A | | A | A | A | ***G*** | A | A | A | G=4.7% 10.8% | GGTAG[A/G]CTGCA | 27568606 | Intron 1 | 72710403 |
| AC | | AC | **--** | AC | AC | AC | AC | AC | --=7.0% 5.1% | CAAAT[--/AC]AGTTA | 27568569 | Intron 1 | 77534147 |
| C | | C | C | C | C | ***G*** | C | C | G=17.0% 25.2% | ACTCT[C/G]TGGTT | 27568180 | Intron 1 | 7875392 |
| G | | G | G | ***C*** | G | G | G | G | C=2.8% 1.9% | CTGGA[G/C]CCATA | 27568133 | Intron 1 | 17769300 |
| C | | C | C | C | C | ***A*** | C | C | A=17.0% 25.2% | GTCAA[C/A]ACTTT | 27567937 | Intron 1 | 7872223 |
| T | | T | G | G | T | G | T | G | T=43.1% 43.8% | GATAT[G/T]TTCAA | 27567637 | Intron 1 | 10967988 |
| G | | G | G | G | G | ***A*** | G | G | A=17.0% 25.2% | TGTGG[G/A]TTGAA | 27567610 | Intron 1 | 10757669 |
| G | | G | G | ***A*** | G | G | G | G | A=2.8% 1.8% | GACCC[G/A]CAGTA | 27567483 | Intron 1 | 41272891 |
| C | | C | C | C | C | ***T*** | C | C | T=17.0% 25.2% | TTATC[C/T]AAATG | 27567147 | Exon 2 | 10757668 |
| C | | C | C | C | C | G | C | G | G=44.0% 49.1% | AAATA[C/G]CATTA | 27566143 | Intron 2 | 2120721 |
| C | | C | C | C | C | C | ***T*** | C | T=27.9% 27.7% | AGTTT[C/T]ATAAT | 27565938 | Intron 2 | 1031153 |
| A | | A | A | A | A | ***C*** | A | A | C=16.2% 25.2% | GGAAA[A/C]AATGG | 27565716 | Intron 2 | 10757667 |
| A | | A | A | G | A | G | A | G | G=45.3% 50.7% | AAAAA[A/G]GGAGC | 27565302 | Intron 3 | 10967986 |
| A | | A | A | T | A | T | A | A | T=19.0% 27.0% | ACAGA[A/T]AAAAA | 27565294 | Intron 3 | 10757666 |
| T | | T | T | T | T | ***G*** | T | T | G=16.2% 25.2% | TTTTT[T/G]ATTTT | 27565246 | Intron 3 | 10812618 |
| AG | | AG | AG | AG | AG | AG | AG | ***--*** | --=16.8% 23.4% | CTTAA[AG/--]AACAT | 27565221 | Intron 3 | 142843265 |
| A | | G | A | A | A | G | G | A | A=40.2% 41.5% | ACTTA[G/A]AAGAA | 27565107 | Intron 3 | 2492816 |
| A | | A | A | A | A | ***G*** | A | A | G=18.8% 25.3% | TTGCG[A/G]TAAGT | 27564417 | Intron 3 | 7859060 |
| T | | T | T | T | T | ***C*** | T | T | C=18.8% 25.3% | TTAAG[T/C]AAAGG | 27564340 | Intron 3 | 7874565 |
| T | | T | T | T | T | ***C*** | T | T | C=16.3% 25.2% | AAACT[T/C]GAACT | 27564257 | Intron 3 | 10812617 |
| A | | A | A | A | A | ***G*** | A | A | G=18.8% 25.3% | ACTCA[A/G]TCAAA | 27564010 | Intron 3 | 7858531 |
| G | | G | G | G | G | G | G | ***A*** | A=16.8% 23.3% | ATAAT[G/A]ACTAG | 27563870 | Intron 3 | 2453555 |
| A | | A | A | A | A | A | A | ***C*** | C=16.9% 23.4% | AAAAA[A/C]CCATC | 27563757 | Intron 3 | 2484319 |
| C | | T | C | C | C | C | T | T | C=42.0% 43.2% | CCAGG[C/T]TGGTG | 27562883 | Intron 3 | 10441712 |
| A | | T | A | A | A | A | T | T | A=40.1% 43.3% | ACTAT[A/T]AAAAT | 27562295 | Intron 4 | 10812616 |
| C | | T | C | C | C | C | T | T | C=40.1% 43.3% | TGTGC[C/T]TATTT | 27562235 | Intron 4 | 10812615 |
| G | | A | G | G | G | A | A | A | G=25.8% 18.1% | AAAAG[A/G]CACTA | 27562080 | Intron 4 | 34366576 |
| C | | C | C | C | C | C | C | ***T*** | T=16.8% 23.6% | CTGTG[C/T]ACTAC | 27561802 | Intron 4 | 2453554 |
| T | | T | T | T | ***C*** | T | T | T | C=4.9% 10.8% | CATCA[T/C]TGAGT | 27561630 | Exon 5 | 17769294 |
| T | | T | T | C | T | T | T | C | C=19.6% 25.2% | AACCC[T/C]ACACA | 27561051 | Int/Exon5 | 774359 |
| T | | T | T | G | T | G | G | G | T=24.8% 21.4% | GCTAT[G/T]AGCTA | 27560967 | Int/Exon5 | 12347222 |
| C | | C | C | C | C | C | T | T | C=43.7% 48.5% | ACATGT/C]AGAGA | 27560596 | Int/Exon5 | 3849944 |
| G | | G | G | G | G | ***A*** | G | G | A=16.1% 25.2% | ACACC[G/A]AAGCT | 27560419 | Intron 5 | 10967985 |
| A | | A | A | A | A | A | ***T*** | A | T=37.0% 28.1% | TACAC[A/T]ACTGA | 27559999 | Intron 6 | 774358 |
| C | | C | C | C | C | T | T | T | C=27.5% 23.3% | GGAAA[T/C]AGAAG | 27559939 | Intron 6 | 28526385 |
| G | | G | G | G | G | G | G | ***A*** | A=16.8% 23.5% | AAAGC[G/A]AAGAC | 27559837 | Intron 6 | 774357 |
| A | | A | A | A | A | A | G | G | A=41.7% 48.3% | AAGTA[G/A]CACTG | 27559735 | Intron 6 | 1565948 |
| T | | T | T | C | T | T | T | C | C=19.4% 25.2% | CTTAA[T/C]TAACT | 27559723 | Intron 6 | 774356 |
| A | | A | A | A | A | ***G*** | A | A | G=16.2% 25.2% | CCTTT[A/G]GTTTA | 27559676 | Intron 6 | 7860526 |
| G | | G | G | G | G | C | C | C | G=27.3% 23.0% | CCATG[C/G]AACTT | 27558920 | Intron 6 | 2297694 |
| A | | A | A | A | A | A | ***T*** | A | T=25.9% 25.8% | AAAAA[A/T]GTCCT | 27558240 | Intron 7 | 10812614 |
| C | | C | C | C | C | T | T | T | C=25.5% 23.1% | AGAAA[T/C]GATCT | 27558188 | Intron 7 | 4879566 |
| A | | A | A | T | A | T | T | T | A=22.4% 21.3% | AAATT[T/A]TTTTT | 27558148 | Intron 7 | 4879565 |
| A | | A | ***T*** | A | A | A | A | A | T=6.2% 5.2% | AAATA[A/T]TTGCT | 27557984 | Intron 7 | 67245195 |
| T | | T | T | T | T | ***C*** | T | T | C=16.2% 25.1% | TATAA[T/C]TGAAA | 27557921 | Intron 7 | 10757665 |
| T | | C | T | C | T | C | C | C | T=22.7% 16.3% | TTTGC[C/T]GTGGC | 27557835 | Intron 7 | 4879564 |
| C | | C | C | C | C | C/A | C | C | A=0.8% 2.0% | ACATG[C/A]CAAAC | 27556833 | Intron 7 | 62538126 |
| A | | A | A | ***C*** | A | A | A | A | C=2.7% 1.8% | AATTA[A/C]ATGAG | 27557603 | Intron 7 | 112616482 |
| T | | T | T | C | T | T | T | C | C=19.9% 25.4% | GATCA[T/C]AGTTT | 27557539 | Intron 7 | 700828 |
| T | | T | T | T | T | ***C*** | T | T | C=16.2% 25.1% | ATCTT[C/T]GTATA | 27557456 | Intron 7 | 10812613 |
| G | | C | G | G | G | C | C | C | G=23.9% 18.2% | GGTAG[C/G]CAGTT | 27557343 | Intron 7 | 17835861 |
| G | | G | G | G | G | G | G/A | G | A=8.5% 6.5% | AAAAA[A/G]CAGAT | 27557119 | Intron 7 | 34670748 |
| T | | T | T | ***A*** | T | T | T | T | A=2.7% 1.8% | ATAGA[T/A]GAAGT | 27557061 | Intron 7 | 111253152 |
| A | | G | A | G | A | G | G | G | A=27.3% 16.0% | ACAAA[G/A]CTTCC | 27556782 | Exon 8 | 1022902 |
| T | | T | T | ***G*** | T | T | T | T | G=2.7% 1.8% | TTTCT[T/G] TCTTT | 27556466 | Intron 8 | 17769246 |
| T | | T | T | A | T | T | T | T/A | A=3.6% 3.2% | TTTTT[T/A]TAAAA | 27556054 | Intron 8 | 188460764 |
| ***A*** | | T | T | T | T | T | T | T | A=8.6% 0.5% | ACTG**A**[T/A]CAGGC | 27556040 | Intron 8 | 76412392 |
| T | | T | T | C | T | C | T | C | C=49.7% 51.0% | TCTTA[T/C]AGGCA | 27555838 | Intron 8 | 10967984 |
| T | | T | T | T | T | ***A*** | T | T | A=12.9% 24.3% | TGCAC[T/A]CCCAA | 27555819 | Intron 8 | 10967983 |
| A | | A | A | ***G*** | A | A | A | A | G=2.7% 1.8% | ACATC[A/G] TAACT | 27555628 | Intron 8 | 60613335 |
| G | | G | ***A*** | G | G | G | G | G | A=7.2% 5.2% | AAAAT[G/A]TTTCA | 27555123 | Intron 8 | 35815580 |
| C | | C | C | T | C | T | T | T | C=22.8% 21.5% | CCAAA[T/C]GGTTG | 27555018 | Intron 8 | 12686452 |
| G | | G | G | G | G | G | T | T | G=43.8% 48.5% | AAATT[T/G] TTCTA | 27554190 | Intron 8 | 10124158 |
| T | | T | T | T | T | ***C*** | T | T | C=16.2% 25.1% | CAAAC[T/C]GGGCA | 27553878 | Intron 8 | 12349820 |
| G | | G | G | G | G | ***C*** | G | G | C=16.2% 25.1% | GAATA[G/C]AGAGC | 27553386 | Intron 8 | 80272464 |
| T | | T | T | ***C*** | T | T | T | T | C=2.7% 1.8% | GATGC[T/C]GGCCT | 27552974 | Intron 8 | 113076260 |
| G | | G | ***A*** | G | G | G | G | G | A=7.1% 5.2% | TACAG[G/A]TGTGA | 27552634 | Intron 8 | 68005046 |
| C | | T | C | T | C | T | T | T | C=20.6% 16.2% | GACTA[T/C]AGGTG | 27552489 | Intron 8 | 4878487 |
| C | | C | ***T*** | C | C | C | C | C | T=5.9% 5.0% | TCCCA[C/T]CTCAA | 27552462 | Intron 8 | 71510499 |
| T | | C | T | T | T | T | C | T | C=30.2% 33.7% | AGGAG[T/C]GGTGA | 27552195 | Intron 8 | 10967981 |
| T | | T | T | T | T | ***C*** | T | T | C=16.2% 25.1% | ATTCG[T/C]TTTGC | 27551929 | Intron 8 | 12347201 |
| ***T*** | | C | C | C | C | C | C | C | T=8.2 0.5% | GTGTA[C/T]CACAC | 27551621 | Intron 8 | 117867610 |
| C | | C | C | C | C | C | C | ***T*** | T=16.5% 22.9% | AGACC[C/T] TTAGG | 27551042 | Intron 8 | 2453565 |
| A | | A | A | A | A | ***C*** | A | A | C=16.2% 25.1% | TATCA[A/C]TCTAT | 27551001 | Intron 8 | 62538125 |
| T | | T | T | ***C*** | T | T | T | T | C=4.7% 1.8% | ATATA[T/C]GCTCG | 27550170 | Intron 9 | 10120735 |
| T | | T | T | G | T | T | G | G | G=47.9% 51.6% | CCTCC[T/G]GGGTT | 27548937 | Intron 9 | 773723 |
| A | | A | A | A | A | ***C*** | A | A | C=18.8% 26.9% | AACCT[A/C]CACCT | 27548929 | Intron 9 | 10967979 |
| C | | G | C | C | G | C | C | C | G=6.6% 15.6% | ACAAT[C/G]TGATA | 27548843 | Intron 9 | 72727512 |
| ***C*** | | A | A | A | A | A | A | A | C=8.5% 0.5% | AAAAA[A/C]CAATG | 27548514 | Intron 10 | 2305045 |
| A | | A | A | ***G*** | A | A | A | A | G=4.6% 1.8% | GCAAT[A/G]ATATT | 27547988 | Exon 11 | 73440933 |
| - | | - | - | ***A*** | - | - | - | - | A=2.6% 1.8% | ATTTC[-/A]AAAAA | 27547926 | Exon 11 | 200583482 |
| T | | G | T | G | T | G | G | G | T=20.8% 16.1% | TCAGA[G/T]TTGCA | 27547315 | Exon 11 | 3739526 |
| G | | G | G | G | G | G | ***A*** | G | A=27.5% 25.9% | ATTTT[G/A]TTGTG | 27546892 | Exon 11 | 13691 |
| A | | A | A | A | A | ***G*** | A | A | G=16.8% 26.9% | TGTCC[A/G]CATCT | 27546830 | Exon 11 | 9103 |
| G | | G | G | ***A*** | G | G | G | G | A=2.5% 1.8% | CAGAA[G/A]CTGAT | 27546489 | 3' flanking | 117189148 |
| C | | C | C | C | C | C | C | ***A*** | A=16.1% 22.8% | GTGAT[C/A]TGTTT | 27545962 | 3' flanking | 700791 |
| T | | C | T | T | T | T | C | T | C=29.5% 31.8% | TTGCA[T/C]AGGTT | 27545547 | 3' flanking | 10812612 |
| C | | C | C | C | C | C | C | ***T*** | T=16.2% 22.8% | AGGAT[C/T]GTATA | 27545469 | 3' flanking | 812858 |
| G | | G | G | ***C*** | G | G | G | G | C=4.6% 1.9% | CAGGA[G/C]ATCGA | 27544000 | 3' flanking | 59797827 |
| T | | T | ***A*** | T | T | T | T | T | A=7.5% 5.2% | TGCAG[T/A]GGCTC | 27543938 | 3' flanking | 6476001 |
| T | | T | T | T | T | T | T | ***C*** | C=18.2% 23.3% | GAGAG[T/C]CATTA | 27543384 | 3' flanking | 3849943 |
| C | | C | C | C | C | C | C | ***T*** | T=18.2% 23.3% | TGCAA[C/T]AAAAG | 27543283 | 3' flanking | 3849942 |

Detailed genetic map of the major European haplotypes of the *C9orf72* locus includes its promoter (1 kb) and its 3' (3.2 kb) regions. The map includes 117 polymorphic sites with a minor allele having a frequency of 1% or more within the European population. The map also includes the Z haplotype SNPs. Haplotype-specific SNPs are indicated in italic bold font and sub-haplotype variations are signified by two alternative nucleotides in the same table cell (*e.g*., rs4879572 in haplotype F). SNP ID corresponds to the dbSNP database ([www.ncbi.nlm.nih.gov/SNP](http://www.ncbi.nlm.nih.gov/SNP)). SNPs that were described in previous profiling of the R haplotype are highlighted by underline. SNP position on chromosome 9 is according to the GRCh38 assembly (http://www.ncbi.nlm.nih.gov/gene). SNP flanking sequence is shown from position -5 to +5 according to chromosome 9 (+) strand. The *C9orf72* gene is located at the (-) strand and therefore the sequence of *C9orf72* transcripts is complementary to the sequences in the table. SNP minor allele frequency (MAF) in the global population (World) and in Europeans (EUR) is indicated according to the 1000 Genomes data (viewed in Ensembl, <http://www.ensembl.org/Homo_sapiens/Search/Results?q=;facet_feature_type=;site=ensembl;facet_species=Human;page=1>).
