# Supplementary material for: Characterization of C9orf72 haplotypes to evaluate the effects of normal and pathological variations on its expression and splicing
Source: PLoS Genet. 2021 Mar 29;17(3):e1009445. doi: 10.1371/journal.pgen.1009445 (PMC8031855; doi:10.1371/journal.pgen.1009445)
Supplement: S3 Table — (DOCX) [file pgen.1009445.s014.docx]

**S3 Table. Haplotype and HR length results of the research participants**

| **Subject ID #** | **Origin** | **Sex** | **Age** | **Healthy or ALS** | **haplotypes** | **HR length** | **Haplotype-HR matching** |
| --- | --- | --- | --- | --- | --- | --- | --- |
| D-540* | Caucasian | female | 51 | *C9orf72* | R, K | 2 | R_d_K_2_ |
| D-312* | Caucasian | male | 55 | *C9orf72* | R, F | 2 | R_d_F_2_ |
| D-883* | Caucasian | male | 61 | *C9orf72* | R, N | 4 | R_d_N_4_ |
| D-184 | Caucasian | female | 67 | *C9orf72* | R, R | 12 | R_d_R_12_ |
| D-836 | Caucasian | male | 68 | *C9orf72* | R, K | 2 | R_d_K_2_ |
| D-007 | Caucasian | female | 60 | *C9orf72* | R, K | 2 | R_d_K_2_ |
| D-805 | Caucasian | male | 66 | *C9orf72* | R, F | 2 | R_d_F_2_ |
| D-733 | Caucasian | male | 57 | *C9orf72* | R, F | 2 | R_d_F_2_ |
| D-338 | Caucasian | female | 56 | *C9orf72* | R, K | 2 | R_d_K_2_ |
| D-431 | Caucasian | female | 59 | *C9orf72* | R, K | 2 | R_d_K_2_ |
| D-850 | Caucasian | female | 59 | *C9orf72* | R, F | 2 | R_d_F_2_ |
| D-478 | Caucasian | male | 53 | *C9orf72* | R, K | 2,~30 | R_d(30)_K_2_ |
| F-545 | Caucasian | male | 27 | *SOD1* | R, P | 12, 5 | R_12_P_5_ |
| S-142 | Caucasian | male | 50 | sporadic | R, F | 8, 2 | R_8_F_2_ |
| S-314 | Caucasian | female | 58 | sporadic | R, J | 7, 6 | R_7_J_6_ |
| S-427 | Caucasian | female | 49 | sporadic | F, F | 2 | F_2_F_2_ |
| H-900 | Caucasian | male | 66 | healthy | P, P | 5 | P_5_P_5_ |
| H-781 | Caucasian | male | 57 | healthy | P, P | 5 | P_5_P_5_ |
| H-112 | Caucasian | male | 54 | healthy | P, P | 5 | P_5_P_5_ |
| H-1116 | Caucasian | female | 50 | healthy | K, K | 2 | K_2_K_2_ |
| H-1011 | Caucasian | male | 51 | healthy | F, F | 2 | F_2_F_2_ |
| H-654 | Caucasian | female | 57 | healthy | R, R | 10, 2 | R_10_R_2_ |
| F-067 | Caucasian | female | 61 | familial | R, R | 22, 12 | R_22_R_12_ |
| F-440 | Caucasian | male | 57 | familial | R, R | 10, 8 | R_10_R_8_ |
| H-289 | Caucasian | female | 54 | healthy | R, R | ND |  |
| H-556 | Caucasian | female | 67 | healthy | R, R | 18, 8 | R_18_R_8_ |
| H-1021 | Caucasian | female | 52 | healthy | R, R | 10, 8 | R_10_R_8_ |
| H-1077 | Caucasian | female | 63 | healthy | R, R | 12, 10 | R_12_R_10_ |
| F-967 | Caucasian | male | 68 | familial | J, J | 6, 5 | J_6_J_5_ |
| H-656 | Caucasian | male | 70 | healthy | F, K | 2 | F_2_K_2_ |
| H-981 | Caucasian | male | 50 | healthy | F, K | 2 | F_2_K_2_ |
| H-351* | Caucasian | male | 61 | healthy | R, F | 12, 2 | R_12_F_2_ |
| H-157 | Caucasian | male | 54 | healthy | P, F(K) | 5, 2 | P_5_F(K)_2_ |
| F-281 | Caucasian | male | 71 | *SOD1* | R, F | 8, 2 | R_8_F_2_ |
| H-923 | Caucasian | male | 58 | healthy | R, K | 10, 2 | R_10_K_2_ |
| H-251 | Caucasian | female | 60 | healthy | R, F | 8, 2 | R_8_F_2_ |
| H-966* | Caucasian | female | 51 | healthy | P, K | 5, 2 | P_5_K_2_ |
| S-1143 | Caucasian | female | 50 | sporadic | P, F | 5, 2 | P_5_F_2_ |
| F-490 | Caucasian | male | 50 | familial | R, F | 12, 2 | R_12_F_2_ |
| F-337 | Caucasian | female | 69 | familial | J, K | 6, 2 | J_6_K_2_ |
| H-951 | Caucasian | female | 61 | healthy | J, K | 6, 2 | J_6_K_2_ |
| H-682 | Caucasian | female | 60 | healthy | J, K | 6, 2 | J_6_K_2_ |
| H-611 | Caucasian | male | 59 | healthy | R, K | 16, 2 | R_16_K_2_ |
| H-1007 | Caucasian | male | 61 | healthy | P, F | 5, 2 | P_5_F_2_ |
| H-593 | Caucasian | male | 63 | healthy | R, P | 12, 5 | R_12_P_5_ |
| H-785 | Caucasian | male | 56 | healthy | N, K | 4, 2 | N_4_K_2_ |
| H-234 | Caucasian | male | 50 | healthy | R, P | 8, 5 | R_8_P_5_ |
| H-1146 | Caucasian | male | 68 | healthy | P, Q | 5, 2 | P_5_Q_2_ |
| H-901 | Caucasian | male | 69 | healthy | R, K | 7, 2 | R_7_K_2_ |
| H-592 | Caucasian | female | 62 | healthy | R, F | 8, 2 | R_8_F_2_ |
| H-957 | Caucasian | female | 56 | healthy | J, Q | 6, 2 | J_6_Q_2_ |
| H-1035 | Caucasian | female | 65 | healthy | P, F | 5, 2 | P_5_F_2_ |
| H-868 | Caucasian | female | 67 | healthy | N, F | 4, 2 | N_4_F_2_ |
| H-70* | Caucasian | female | 27 | healthy | P, K | 5, 2 | P_5_K_2_ |
| FOR002 | Mediterranean | male |  | healthy | P, K | 5, 2 | P_5_K_2_ |
| OL | Mediterranean | male |  | healthy | J, K | 6, 2 | J_6_K_2_ |
| HES-1 | Indonesian | female |  | healthy | R, J | 8, 2 | R_8_J_2_ |
| HEK293 | Caucasian? | female |  | healthy | N, Q | 4, 2 | N_4_Q_2_ |

The H-prefix represents healthy individuals, and the D-prefix represents C9-ALS patients. The F and the S prefixes represents familial and sporadic ALS patients, respectively, without *C9orf72* mutation. Asterisks represent fibroblast cultures that were reprogrammed into iPSCs. The HR length was determined by PCR and the length of the R_d_ alleles was not identified. FOR002 and OL are foreskin lines and HES-1 is a human embryonic stem-cells line. The HEK293 line is hypotriploid and the ratio between the Q and N allele is ~2:1. ND: not determined.
