# Supplementary material for: Characterization of C9orf72 haplotypes to evaluate the effects of normal and pathological variations on its expression and splicing
Source: PLoS Genet. 2021 Mar 29;17(3):e1009445. doi: 10.1371/journal.pgen.1009445 (PMC8031855; doi:10.1371/journal.pgen.1009445)
Supplement: S4 Table — (DOCX) [file pgen.1009445.s015.docx]

**S4 Table. RNA-seq reads of the *C9orf72* gene in iPSCs and fibroblasts from control and C9-ALS patients**

| **Cells** | **C9 ALS** | **Intron 1 reads** | **Intron 2 reads** | **Exons 2-5 reads** | **Total reads** | **Exons 2-5/ total reads** | **Intron 1/ Exons 2-5** | **Intron 2/**  **Exons 2-5** |
| --- | --- | --- | --- | --- | --- | --- | --- | --- |
| H-351 iPSC | _ | 254 | 21 | 455 | 80.8M | 5.6e-6 | 0.558 | 0.046 |
| H-70 iPSC | _ | 224 | 20 | 371 | 70.0M | 5.3e-6 | 0.604 | 0.054 |
| H-966 iPSC | _ | 150 | 10 | 275 | 61.8M | 4.4e-6 | 0.545 | 0.036 |
| **Control iPSC** | _ |  |  |  |  |  | **0.569** | **0.045** |
| D-883 iPSC | + | 1591 | 108 | 905 | 96.5M | 9.4e-6 | 1.758 | 0.119 |
| D-312 iPSC | + | 1779 | 122 | 967 | 93.1M | 1.0e-5 | 1.840 | 0.126 |
| D-540 iPSC | + | 1800 | 127 | 1042 | 83.1M | 1.3e-5 | 1.727 | 0.122 |
| **C9-ALS iPSC** | + |  |  |  |  |  | **1.775** | **0.122** |
| H-1143 Fib. | _ | 8 | 2 | 49 | 23.2M | 2.1e-6 | 0.163 | 0.041 |
| H-351 Fib. | _ | 10 | 2 | 45 | 25.2M | 1.8e-6 | 0.222 | 0.044 |
| **Control Fib.** | _ |  |  |  |  |  | **0.192** | **0.042** |
| D-883 Fib. | + | 49 | 7 | 67 | 23.2M | 2.9e-6 | 0.731 | 0.104 |
| D-312 Fib. | + | 64 | 10 | 70 | 27.3M | 2.6e-6 | 0.914 | 0.143 |
| D-184 Fib. | + | 107 | 11 | 100 | 28.5M | 3.5e-6 | 1.070 | 0.110 |
| **C9-ALS Fib.** | + |  |  |  |  |  | **0.905** | **0.119** |

Intron 1 refers to the number of reads that align to the region between exon 1b and exon 2. Exons 2-5 refer to the number of reads that align to exons 2-5 of V2 and V3. Intron 2 is about 6-fold shorter than intron 1. Fib: Fibroblasts; M = million.
