# Supplementary material for: Characterization of C9orf72 haplotypes to evaluate the effects of normal and pathological variations on its expression and splicing
Source: PLoS Genet. 2021 Mar 29;17(3):e1009445. doi: 10.1371/journal.pgen.1009445 (PMC8031855; doi:10.1371/journal.pgen.1009445)
Supplement: Attachment — Submitted filename: Reply to Editor PLOS Genetics 26-Jan.docx [file pgen.1009445.s017.docx]

**26.1.21**

Professor Giorgio Sirugo, MD, PhD

Professor Hua Tang, PhD

Editors

PLOS Genetics

**Re: "Characterization of C9orf72 haplotypes to evaluate the effects of normal and pathological variations on its expression and splicing" (PGENETICS-D-20-01137)**

Dear Editors**,**

We wish to thank you and the reviewers for evaluating our manuscript, and for providing us the opportunity to revise it. Your constructive comments furnished valuable insights to improve the content of the manuscript. In the enclosed revised manuscript, we addressed all the comments that were raised by the reviewers. We hope that you will find the revised manuscript suitable for publication in PLOS Genetics.

**Sincerely,**

**Benjamin Reubinoff, M.D. PhD**

Point by point response to the reviewers' comments

**Reviewer #1**
*"The biggest problem is the lack of focus on the purpose of this study. The main objective is not conveyed to the reader."*

*"The search for pathologically increased HR-specific haplotypes would be significant…. In the end, they ended up analyzing normal haplotypes within the normal range… It is certainly worthwhile to understand the actual situation, but if that is the purpose of the study, I think it would be better to focus the content on it."*

**…**" *The first half of the paper and the second half of the paper even seem to be dissociated. It could be said that this paper has analyzed the haplotypes of the normal allele in detail and used the results to examine the effect of the allele on the expression of the same gene in a sample of ALS patients who were heterozygous. However, the paper is poorly structured and does not adequately convey its significance to the reader. I think it would be better to significantly change the structure of the paper and focus on a clear objective."*

We thank the reviewer for this comment and have revised the manuscript structure to highlight the focus and flow of the results. In the revised version, we emphasized that the main goal of the study was to analyze *C9orf72* expression and splicing from the R_d_ allele compared to the complementary normal non-R allele in ALS patients. Towards this end, we created a detailed molecular map of SNP signature and HR length of R and other *C9orf72* haplotypes in Caucasians. This map enabled us to determine the allelic origin of transcripts per patient, and to examine the effects of pathological HR length on *C9orf72* expression and splicing*.*

We revised the abstract, author summary, and the main text of the manuscript along this line and the major main text revisions are marked in red. We also shortened the length of the manuscript to deliver a more concise and focused message.

The section containing "Sanger sequencing can determine the relative allelic expression" was omitted from the main manuscript, and mostly transferred to the Supplementary Materials, including Fig 2, which was integrated into S3 Fig.

Most of the details in Table 1 in the original manuscript were transferred to S2 Table.

Table 2 in the original manuscript was transferred and incorporated into S4 Table.

We reduced the length of the section in the Results regarding the effects of K haplotype and HR length (in the normal range) on intron 1 levels. We transferred Fig. 3E and F to the supplementary data (as S5 Fig). In the Discussion section, the two possible mechanisms regarding the retention of intron 1 that originates from the K allele were removed from the main text to the Supplementary Materials of the revised manuscript as S10 Fig.

"*The part that examines the effect of this augmented allele on transcription is also abrupt because of the flow of this paper; I think it is necessary to show how much of the V1 and V3 allele is expressed to the V2 allele, and then discuss whether or not it affects the pathology. As currently described, it is unclear what the significance of this change is. Thus, in conclusion, this result may be useful in the ASO setting, but it is unclear what significance the effect on transcription will have."*

In line with the reviewer's comment, we performed an RNA-seq experiment to evaluate the absolute levels and ratio of V1, V2 and V3 of *C9orf72* transcripts in control and C9-ALS patients. However, there was a bias against exon 1 of *C9orf72,* and its read numbers were relatively low. A similar bias was previously reported by Sareen *et al*., Sci Transl Med, 2013). Therefore, we could not accurately determine the absolute contribution of each variant in healthy controls and C9-ALS patients.

In the literature, V2 accounts for 85-95% of the *C9orf72* transcripts in healthy individuals (Liu *et al*., 2013; Sareen *et al*. 2013). We added this information to the Introduction section of the revised manuscript (lines 106-7). It was shown that there is a significant reduction in V2 transcript levels (Shi *et al*., 2018; Liu *et al*., 2014; Xi *et al*., 2013), and consequently C9orf72 protein (Waite *et al*., 2014; Sivadasan *et al*, 2016) in C9-ALS patients compared to healthy individuals. It was suggested that the reduction in C9orf72 protein contributes to the pathogenesis of the disease (Shi *et al*., 2018; O'Rourke *et al*., 2016; Sivadasan *et al*., 2016).

In our study, we shed light on the allelic origin of the reduction of V2 transcripts in C9-ALS. By using our novel platform of haplotype molecular mapping combined with Sanger sequencing, we showed the relative significant reduction in R_d_-originating V2 transcripts compared to the complementary healthy allele. This finding contributes to the understanding of the overall reduction in V2 transcripts in C9-ALS patients. Moreover, this platform may be valuable for analyzing allele-specific transcriptional effect of new candidate drugs. For example, in the case of a drug that enhances V2 transcripts, it will be possible to differentiate between expression augmentation from the healthy versus diseased allele. This issue is highlighted in the revised manuscript in the Discussion section (lines 539-42).

In addition, according to the reviewer's request, in the revised Discussion section we indicate the potential meaning of our findings regarding variant expression and splicing of transcripts from R_d_ allele to the disease pathogenesis. We note: " Given the reduced V2 levels from R_d_ compared to R_h_ alleles, the similar V1 and V3 levels, combined with the high levels of cryptic and unspliced intron 1 transcripts from the R_d_ allele (Fig 4C), it appears, as previously noted (Liu *et al*., 2013; Sareen *et al*. 2013), that HR expansion in C9-ALS patients is associated with a marked shift in R_d_ allele promoter utilization from the downstream harmless promoter to the upstream and potentially harmful promoter." (487-92).

**Reviewer #2:**

Major comments

*"The authors did not indicate whether they obtained a signed consent for the analysis of their genetic characteristics.*"

The ALS patients and the healthy control participants signed an informed consent that included genetic analysis of acquired biospecimens. This is clarified in the revised manuscript in the Method section. (lines 556-9).

*"Furthermore, it would be interesting to have more information about these* *Americans of European descent: are the authors certain that they can consider them as "Europeans" especially if they have been Americans for several generations? If this is not possible please discuss this in the results and discussion sections."*

We thank the reviewer for his comment. Accordingly, in the revised manuscript we omitted the term "Americans of European descent" and defined the participants as "Caucasian Americans".

*"On page 8, the authors indicate that they have identified a "Z" haplotype, if I understand this discovery was made by another team or study. If this is the case please cite the reference or origin. I don't think this is somewhere in the manuscript."*

The "Z" haplotype with its unique three SNPs was not previously defined. Among Asian C9-ALS patients, genotypic analysis showed that both alleles did not match the R haplotype. It was deduced that the diseased allele originated from a founder other than the one bearing the R haplotype. Nevertheless, these studies, cited in our manuscript, did not characterize the non-R diseased haplotype and its tag SNPs (He *et al*., 2015; Chen *et al*., 2016; and Shamim *et al*., 2020). We independently identified the Z haplotype and defined its unique tag SNPs in healthy individuals. Based on the genotyping results in the abovementioned articles, we were able to conclude that the Asian risk haplotype is the "Z" haplotype. We clarify this point in the revised manuscript (lines 443-50).

*Could the author indicate if the patients studied on page 11 (called "European subjects": 15 and 30 subjects) are the same as those used for the description of haplotypes?*

Yes, the patients studied on page 11 are the same as those used for the description of haplotypes. To avoid misunderstanding, we revised the manuscript as follows:

" We therefore analyzed the HR length in Caucasian American subjects for whom we had identified their haplotype, as described above (Fig 1A, left chart) " (lines 220-1).

*The authors used (RT-)PCR/Sanger sequencing for gene expression studies. Please indicate on page 12 that these results were obtained on the transcripts and therefore are studies on RT-PCR products.*

We thank the reviewer for his helpful comment. We therefore revised the text as follows:

"To determine the relative allelic contribution to RNA transcripts we used our SNP haplotype mapping for peak height analysis of heterozygous SNP sites in Sanger sequencing chromatograms of RT-PCR products."(lines 255-7).

According to this comment, we performed similar revisions at a few other locations throughout the manuscript and the supplementary data (lines 301, 307, 350, 394, 625 and S1 Table).

*The authors have purified their RT-PCR products to set up the technique and to produce Figure 2B. Was the purification step always performed for the study of patients and healthy individuals? Why did the authors not use allele-specific RT-PCR or RNA-seq for this work?*

Yes, to achieve reproducible results with low background, we purified the PCR/RT-PCR fragments for both patients and healthy individuals before Sanger sequencing.

Allele-Specific RT-PCR (ASPCR) is based on three primers. One primer is common for both alleles and might be optimal in its sequence and location (optimal Tm and GC content, low secondary structure, *etc*). The two other primers are discriminative primers that differ in their last (3') nucleotide. ASPCR works because the primer that forms a 3′ mismatch with the DNA template will be refractory to primer extension by Taq polymerase.

The problem with this method is that one cannot choose the location of the discriminative primers. Thus, in ~90% of the cases, the primer sets are not optimal. In addition, the alternative last nucleotide is usually C/T or A/G (A/T or G/C are relatively rare). Thus, the alternative nucleotide may change the annealing temperature of the primer.

In contrast to the ASPCR, the Sanger method does not usually restrict the positions of primers to a specific site, and accordingly is applicable for almost every SNP site.

Regarding the RNA-seq: we performed RNA-seq analyses, and the results are described in Fig 3 A-B and in S4 Table.

However, it should be noted that there are three major problems with allele-specific RNA-seq analysis:

- Low coverage for most introns;
- Due to the short length of the reads (74 or less), co-localization of heterozygous SNP site with splice site junction is infrequent;
- In *C9orf72*, there is a bias against the 5' end of the transcripts, and the first exon of V1-V3 is underrepresented. A similar observation was described previously by Sareen *et al*.: "sampling of exons 1a and 1b was relatively low in the RNA-seq reads, possibly due to the high GC content and repetitive sequence in this region" (Sareen *et al*., 2013).

*I would like to know if this relative quantification technique by RT-PCR/Sanger is reproducible: for example, are the peak heights (ratios) the same if the experiment is repeated three times on the same RNA sample?*

Yes, there are only minor differences in the peak height ratios in both genomic DNA and cDNA analyses, and the results are reproducible and reliable as in S3 Fig.

*I propose that the authors reduce the size of the section "HR length and haplotype effects on introns 1 and 2 levels".*

We reduced the size of this section by moving methodological details to the Methods section and incorporating Table 2 and its data from the original manuscript into S4 Table in the supplementary.

In addition, Fig. 3E and F were transferred to the supplementary data (as S5 Fig).

We also reduced the length of the text regarding the effects of K haplotype and HR length (in the normal range) on intron 1 levels.

*page 17: is the "heterozygous SNP site" cited in the second paragraph of page 7 present on transcripts V1, V2 and V3, or only V1 and V3?*

We used the same heterozygous SNP sites for the analysis of V1+V3 and for V2. One of these SNPs is rs10757668 that was cited in page 7 and shown in Fig. 2A.

To clarify this issue, we revised the wording in the manuscript: "We next evaluated the relation between HR length and V2 expression in fibroblasts by using the same methodology and heterozygous SNP sites (Fig 2A and S1 Table#30-32)" (lines 277-9).

*Did the authors investigate whether the use of a cryptic donor splice site resulted in a change of the acceptor splice site?*

We thank the reviewer for raising this comment. We indeed evaluated whether using a cryptic splice site changed the acceptor splice site. In the vast majority of cases (23 out of 24 evaluated cryptic splicing events), the acceptor site was not changed and the cryptic exon 1 joined to the 5'-end of exon 2 at the authentic acceptor site. This point is now clarified in the revised manuscript:

"In the three control iPSCs, we identified a single read containing cryptic splice junction. In contrast, in the three C9-ALS iPSCs, we identified 24 reads containing a cryptic splice junction, and all but one joined to the 5'-end of exon 2 (Fig 4C and S9 Fig)" (lines 423-6).

*It would be important that the authors indicate in the discussion section that the observations they made should be replicated in neurons, or (better) motor neurons such as motor neurons derived from iPSCs of patients or healthy individuals. Indeed, the expression levels of the transcription variants could be very different compared to fibroblasts or iPSCs, isnt’it? Same for cryptic sites.*

We thank the reviewer for his comment and revised the discussion accordingly:

"Notably, our results regarding *C9orf72* transcripts and cryptic splicing were observed in fibroblasts and iPSCs, and it will be important to evaluate the generalizability of the results in MNs and more preferably MNs derived from iPSCs of ALS patients" (lines 516-18).

Minor comments:
*Page 12: We aimed to determine the possible effects of haplotype and HR length on C9orf72. Perhaps “relations” could be better than “effects”, as this work does not allow us to observe the mechanistic effects.*

We revised the manuscript according to the reviewer's comment and replaced along the manuscript the words "effect" and "affect" with the words "associated with" or "relations". For example, see the section titles at lines 243 and 320.

*Fibroblast cultures: could the authors indicate the passage number of the cultures. The age of the cultures has an impact on the functioning (gene expression) of the cells.*

We added the passage number of fibroblasts and iPSCs to the Methods section as follows: "Total RNA was isolated from fibroblasts (passages 4-7) and iPSCs (passages 6-10) using…" (line 600).

*I propose to place Table 2 in the Supplementary Data (it is fully described in the text).*

According to the reviewer's suggestion, we integrated Table 2 into S4 Table.

*Figure 1: replace “Our Sample” by “American European population (our sample)”*

We replaced "Our Sample" with "Caucasian Americans (our sample)".

**Reviewer #3:**

*This manuscript reports a rather thorough study of C9orf72 haplotypes in the transcribed region, in relation to length of the hexanucleotide repeat (HR), and the effect of length of repeat on expression at the RNA level in fibroblast, in iPSCs of ALS patients or controls (n=3 for each). The authors investigated alternate promoter use, intron retention and activation of cryptic donor splice sites distal to HR in intron one. In all of these, they could use the haplotype specific SNPs in the transcript they have identified in their haplotype analysis to distinguish expression from each allele.
However, I thought the paper quite lengthy, and the importance of the observations for the pathogenic mechanisms in ALS rather unclear or confirmatory (the founder effect) in some cases.*

*For instance, the effect of number of repeats on V1+V3 expression is very striking in the normal range (Fig3B) but what may be the relation to ALS mechanisms, as the Rd alleles have actually lower (but non significantly) relative expression than the Rh (7-16 repeats (Fig3D).*

According to reviewer's comment we shortened the length of the manuscript to deliver a more concise and focused message.

The section containing "Sanger sequencing can determine the relative allelic expression" was omitted from the main manuscript, and mostly transferred to the Supplementary Materials, including Fig 2, which was integrated into S3 Fig.

Most of the details in Table 1 in the original manuscript were transferred to S2 Table.

Table 2 in the original manuscript was transferred and incorporated into S4 Table.

We reduced the length of the section in the Results regarding the effects of K haplotype and HR length (in the normal range) on intron 1 levels. We transferred Fig. 3E and F to the supplementary data (as S5 Fig). In the Discussion section, the two possible mechanisms regarding the retention of intron 1 that originates from the K allele were removed from the main text to the Supplementary Materials of the revised manuscript as S10 Fig.

We thank the reviewer for noting our observation of the striking effect of HR length, within the normal range, on the activity of the upstream promoter of V1+V3. From the perspective of C9-ALS/FTD disease, reduced levels of functional C9orf72 protein are suggested to play a major role in disease pathogenesis (Shi *et al*., 2018; O'Rourke *et al*., 2016; Sivadasan *et al*., 2016). The V1+V3 expression levels from the healthy allele, in C9-ALS/FTD patients, may be important because they contribute to overall levels of normal *C9orf72* transcripts, which are translated to functional C9orf72 protein. To strengthen this point, we added to the revised manuscript results regarding the overall allelic *C9orf72* expression in short versus moderate HR lengths. Like the effect on V1+V3 expression, moderate HR length was associated with higher overall allelic *C9orf72* expression compared to short HR length (lines 281-5; Fig 2D). Hence, elevated *C9orf72* expression from the normal allele with moderate HR lengths may mitigate reduced *C9orf72* expression from the R_d_ allele, which is commonly observed in C9-ALS/FTD patients. On the other hand, the increased upstream promoter activity of the R_h_ allele is associated with production of pre-spliced RNAs with a moderate HR length. These pre-spliced transcripts may contribute to abnormal G-quadruplex structures, liquid phase separation, and other abnormalities that originate from the pathologically expanded HR allele. Thus, a moderate length of the HR in the complementary allele may exert a double-edged sword effect, with positive and negative impact on the disease phenotype.

In the Results section of the revised manuscript, we indicate the importance of *C9orf72* expression levels from the non-mutated allele of C9-ALS/FTD patients (lines 244-9).

In the revised Discussion section, we discuss the possible positive and negative effects of increased V1+V3 expression (and the overall allelic expression of *C9orf72*) from the non-mutated allele of C9-ALS/FTD patients (lines 471-81).

*One positive aspect is certainly that the identification of transcribed SNPs specific for the R haplotype will allow to study better in an allelic specific way the cis effect of expansion in patient samples.* *As I am not a specialist of c9orf studies, I will ask a question that may be valid or not, but I think it would be interesting to have the answer of the authors. All the abundant c9orf literature concentrate on the effect of HR expansion on expression of the c9orf72 gene at RNA or protein level (DNA methylation, toxic RNA, toxic dipeptides..). However, there are 2 nearby genes INFK and MOB3B whose promoters are at about 50 or 44kb from the HR. There are several instances of intronic SNPs that actually affect the expression of the nearby gene for instance FTO/IRX3, Smemo et al Nature 2014, Lactase/MCM6 Labrie et al Nature Struct Mol Bio 2016. Were there any studies testing the potential effect of HR expansion on expression of these two genes. As the at-risk haplotype extends in most cases to the transcribed regions of INFK and MOB3B, would it be possible to look in the same allelic specific way to the effect of HR length in normal or expanded alleles?*

*Would the RNAseq experiments carried here allow to test this hypothesis?*

We thank the reviewer for raising this important issue.

*C9orf72*-knockout mice express normal *IFNK* and *MOB3B* transcript levels, indicating that in contrast to *FTO-IRX* and *MCM6-LCT* relationships, the *IFNK* and *MOB3B* genes are not regulated by *C9orf72* sequences in mice, and probably neither in humans (Atanasio *et al*., Sci. Rep. 6, 1-14, 2016). Yet, as the reviewer suggests, it is possible that expression of the two genes is altered in C9-ALS/FTD patients.

Mammalian genomes are organized into a hierarchy of megabase-scale Topologically Associating Domains (TADs) and the tandem repeats in *C9orf72, HTT* (Huntington’s disease), *DMPK* (Myotonic dystrophy 1), *FMR1* (fragile X syndrome), and *FXN* (Friedreich’s ataxia) are co-localized with TAD boundaries (Sun JH *et al*., Cell 175, 224–38, 2018). Thus, their repeat expansion may potentially change the 3D organization and alter gene expression regulation.

In *HTT* and *DMPK*, the repeat expansions do not change chromatin organization (Gustavo *et al*., Science Advance 6, 6-17, 2020). However, in *FMR1* and *FXN* there are dramatic changes in the 3D organization (Sun JH *et al*., Cell 175, 224–38, 2018; Chan *et al*., Hum. Mol. Genet. 22, 2662–75, 2013). A possible effect of *C9orf72* HR expansion on chromatin 3D organization was not studied.

As the reviewer suggested, it is possible, in essence, to use Sanger sequencing or RNA-seq to determine the relative expression of the two alleles of *IFNK* and *MOB3B* genes. Notably the IFNK gene is selectively expressed in keratinocytes, monocytes, and dendritic cells, and enhanced upon viral exposure. However, its expression in iPSCs and fibroblasts is very low, and the gene is only 2 kb long with very few heterozygous SNP sites. Therefore, in practice, our RNA-seq data are inadequate to study the allelic expression of IFNK. Moreover, haplotype SNP molecular mapping of these genes is required for both Sanger and RNA sequencing allelic analysis. Given the extensive data that is included in our manuscript, we decided that further analysis of the effect of HR length on *IFNK* and *MOB3B* genes expression would be more appropriate for a separate study.

*A second also naïve questions: regarding the founder effect: there are different possible scenarios. The longer repeat length around 8 may be by itself sufficient to generate in a highly recurrent way larger alleles that themselves become more highly unstable, and one would thus not expect any major sub-haplotypes in patients. However, an alternate possibility is that of a single or rather small number of events that generated a longer repeat, for instance 12 to 15, and these secondary alleles are really the precursors: in this case one could expect to have a few rarer SNVs (too rare to be studied by standard SNP array strategy) in patients, associated to the haplotype carrying the pathogenic expansions. Was this investigated? Would the authors data allow to test this hypothesis?*

Generally, in humans, there is about one mutation per 10^8^ bases per generation, and about a 0.1% chance to acquire a new point mutation along the 110 kb R_d_ allele per generation. Thus, the chance for a unique SNV that is common to all or most of the C9-ALS R_d_ alleles, but none of the normal R alleles, is very low in every evolutionary scenario. Nevertheless, despite the low chance, we used our data and looked for a unique R_d_ allele-specific SNP along the 30kb *C9orf72* locus, but unfortunately, we did not find such a SNP.

*The authors state in abstract, author summary, and twice in discussion (p25, where there is a bit of redundancy), including the last sentence, that the R haplotype characterization may be invaluable or pave the way for allele specific therapeutic strategies. However, given the huge difference in length of the pathogenic repeat versus nonpathogenic, would a* *repeat based targeting strategy be actually simpler (or would it suffer from difficulties linked to the GC only sequence?)*

We thank the reviewer for raising the possibility of repeat-based targeting strategy. This strategy was evaluated in a previous manuscript (Donnelli *et al*., Neuron 80, 415-28, 2013). In this paper, ASO designed against the HR sequence, efficiently reduced the number of nuclear RNA foci generated by the expanded HR of the *C9orf72* mutated gene. Nevertheless, Vourch *et al*. (Neurobiol. Aging, 2021) showed that there are GGGGCC repeats in 74 additional genes, and some of them they are within the first exon. Thus, an ASO that targets the pathogenic repeat may reduce the expression of multiple genes. Moreover, due to its 100% GC content, it may bind with high affinity to sequences that differ from the target sequence by ~1-3 nucleotides.

This issue is discussed in the revised manuscript (lines 523-6).

*Fig4 Did the authors investigate whether intron retention is modified when the iPSCs are differentiated to neuronal precursors or neurons?*
We thank the reviewer for his comment and agree that the data we obtained in C9-fibroblasts and iPSCs should be further confirmed in C9-iPSC-derived motor neurons.

To address this point, we induced MN differentiation using our published protocol (Ben-Shushan *et al.*, Stem cells 33, 403-15, 2015). However, the MN cultures that we obtained were not consistently homogenous and their RNA quality was variable. Given these difficulties and the extensive data that is currently included in the study, it appears that this analysis is more suitable for a separate study.

We address this point in the revised discussion as follows:

"Notably, our results regarding *C9orf72* transcripts and cryptic splicing were observed in fibroblasts and iPSCs, and it will be important to evaluate the generalizability of the results in MNs and more preferably MNs derived from iPSCs of ALS patients." (lines 516-8).

*Editorial suggestions
Table 1 takes 2 pages and details all the tested SNPs in respect to the 8 haplotypes. However, for the vast majority of researchers working on c9orf because of its link with ALS; the table in the main text could be reduced to those SNPs specific for the R haplotype and the rest could be in a supplementary table.*

We thank the reviewer for raising this point and agree that the length of the Table (two pages) is too long. We therefore transferred the data regarding the position of the SNPs, their flanking sequence and their frequency in the global population and in Europeans to S2 Table. This enabled us to shorten Table 1 to a single page.

Yet, we thought that presentation of the SNPs of all eight haplotypes in a single table may be beneficial to the reader (and potentially for the design of personalized ASO treatment) since the differentiation between R and the complementary allele is based on the analysis of the SNPs in both alleles.

For example, SNP rs2282241-**C** (in intron 1) is shared by R, K, N and J haplotypes, while SNP rs2282241-**A** is shared by F, P, Q and Z haplotypes. Hence, in ALS patients with R_d_F, R_d_P, R_d_Q, and R_d_Z diplotypes, this SNP will be heterozygous and appropriate for differentiation between the alleles, as opposed to patients with R_d_K, R_d_N, and R_d_J that are homozygous in this nucleotide.

We clarify this issue in the Discussion section of the revised manuscript (lines 531-3).

*Also, I counted in the table 6 R specific SNPs, while there seems to be 10 or 11 in Fig 1B: what is the reason for this discrepancy?*

In Fig 1B, indeed there are 12 dots for R-specific SNPs. In the original manuscript Table 1 referred to the transcribed region of *C9orf72,* while S2 Table referred to the 5' and 3' flanking regions of the gene. Thus, the dots represent the R-specific SNPs included both Table 1 (7 SNPs) and S2 Table (5 SNPs). To simplify this issue, in the revised manuscript S2 Table contains now all the analyzed SNPs at the transcribed region of *C9orf72* and the 5' and 3' flanking regions of the gene, hence all the 12 R allele's SNPs are included in a single Table. This point is clarified in the legend of Fig 1B "Frequency of haplotype-specific SNPs along the transcribed region of *C9orf72* gene and its 5' and 3' flanking regions among Europeans (S2 Table)." (lines 207-8).

*Finally, it took me some effort to try to correlate to the R haplotype specific SNPs described previously by Smith BN et al (2013) or Goldstein O et al Neurobiol Aging 2018, not cited). It seems that most or all of these previous SNPs fall outside the region investigated here and thus of the transcribed region? I suggest that either in the shortened table 1 or in Fig1a the authors position these previous R specific SNPs on the map.*

We thank the reviewer for this suggestion, and accordingly we added in Table 1 and S2 Table an underline to SNPs that were previously used to characterize the R haplotype.

In the revised manuscript, we cite Goldstein O. *et al*., Neurobiol Aging, 2018. (line 118; reference#25).

*Fig2 is a control of validity for a rather standard methodology, and it could be in supp info, as most of page 12.*
The section regarding "Sanger sequencing can determine the relative allelic expression" was transferred to the Supplementary Materials, including Fig 2, which was integrated into S3 Fig of the revised manuscript*.*

*The effect of K haplotype is described in 1 page in results, and half a page in discussion. While the data are convincing, is there any hint that this could be relevant to ALS risk? Are there studies in ALS that might point to an effect on risk either in trans to an expanded repeat, or as a risk factor for sporadic ALS without expanded allele. Otherwise, the length given to this perhaps anecdotic observation seems too large.*

The K haplotype is not a known risk factor for familial or sporadic ALS/FTD diseases. However, the retention levels of intron 1 that originate from R_d_ allele are analyzed relative to the levels originating from the complementary normal allele. Therefore, it was important to study the splicing efficiency of the various normal alleles.

According to the reviewer's request, we reduced the length of the section in the Results regarding the effects of K haplotype and HR length (in the normal range) on intron 1 levels. We transferred Fig. 3E and F to the supplementary data (as S5 Fig). In the Discussion section, the two possible mechanisms regarding the retention of intron 1 that originates from the K allele were removed and are presented in the Supplementary Materials of the revised manuscript as S10 Fig.

.
